# Increased expression of miR-93 is associated with poor prognosis in head and neck squamous cell carcinoma

**DOI:** 10.1007/s13277-015-3038-6

**Published:** 2015-01-13

**Authors:** Guo Li, Shuling Ren, Zhongwu Su, Chao Liu, Tengbo Deng, Donghai Huang, Yongquan Tian, Yuanzheng Qiu, Yong Liu

**Affiliations:** 1 Department of Otolaryngology Head and Neck Surgery, Xiangya Hospital, Central South University, Xiangya Road 87, Changsha, 410008 Hunan China; 2 Otolaryngology Major Disease Research Key Laboratory of Hunan Province, Changsha, 410008 Hunan China

**Keywords:** Head and neck squamous cell carcinoma, MicroRNA-93-5p, In situ hybridisation, Metastasis, Prognosis

## Abstract

MicroRNA-93-5p (miR-93) is a novel oncogenic microRNA (miRNA) and is elevated in diverse human malignancies. Aberrant expression and dysfunction of miR-93 are involved in many types of human tumours. However, the exact role of miR-93 remains unclear in head and neck squamous cell carcinoma (HNSCC). The objective of this study is to determine the expression pattern and clinical significance of miR-93 in HNSCC. MiR-93 expression levels in 103 primary HNSCC tissues and 16 corresponding non-cancerous epithelia were analysed by miRNA in situ hybridisation and correlated with the clinicopathological parameters and patient outcomes. Moreover, the expression of miR-93 was examined in four HNSCC cell lines and 17 pairs of HNSCC tissues and their corresponding adjacent tissues using quantitative real-time PCR (qRT-PCR). The miR-93 levels in HNSCC tissues and cell lines were significantly higher than those in the non-cancerous tissues. Notably, high miR-93 expression was significantly associated with T classification, lymph node metastasis and clinical stage. Kaplan–Meier survival analysis demonstrated that patients with high miR-93 expression had poorer overall survival than patients with low miR-93 expression. Multivariate Cox regression analysis revealed that miR-93 overexpression and lymph node metastasis were independent prognostic factors in patients with HNSCC. This study demonstrated that miR-93 expression was significantly increased in HNSCC tissue samples and cell lines and that miR-93 overexpression was associated with tumour progression, metastasis and poor prognosis in HNSCC patients. These results suggest that miR-93 may play a critical role in the initiation and progression of HNSCC, indicating that miR-93 may be a valuable marker for the prediction of metastasis and prognosis in HNSCC.

## Introduction

Head and neck cancer, one of the most harmful and life-threatening diseases, accounts for 6 % of all cancers in humans [1]. Head and neck squamous cell carcinoma (HNSCC) is an important pathological type of head and neck cancer. Despite progress in conventional therapies, such as surgery, radiotherapy and chemotherapy, the survival rate in patients with HNSCC remains unsatisfactory, and metastasis is the major cause of poor prognosis in patients with HNSCC [2]. Therefore, identifying new specific markers of metastasis would be of great value for the treatment and prognosis of HNSCC.

MicroRNAs (miRNAs) are non-coding small RNAs that contain approximately 19–25 nucleotides [3]. They are transcribed from genomic DNAs to generate long primary transcripts, followed by modification by the RNase III-type enzymes Drosha and Dicer to produce pre-miRNAs and mature miRNAs [4]. Mature miRNAs posttranscriptionally regulate the expression of target genes by directly binding to their 3′-untranslated regions (3′-UTR) [5]. As a new class of regulatory molecules, miRNAs serve vital functions in many physiological and pathological processes, particularly carcinogenesis, and of these miRNAs, several function as oncogenic miRNAs, whereas others are tumour suppressors [6]. Moreover, differentially expressed miRNAs in HNSCC have been screened, and several miRNAs were identified as specific biomarkers to evaluate lymph node metastasis and prognosis in patients with HNSCC [7, 8].

MiRNA-93-5p (miR-93) is from the miR-106b-25 cluster, a paralogue cluster of the miR-17-92 cluster. Located in intron 13 of the host gene MCM7 at chromosome 7q22, the miR-106b-25 cluster consists of the highly conserved miR-106b, miR-93 and miR-25 [9]. MiR-93 is differentially expressed in a variety of cancers, including cancers in the lung, stomach, colon, liver and breast [10–14]. Differential expression of miR-93 and its correlation with metastasis have also been confirmed in primary osteosarcoma cells [15]. Another study found that overexpression of miR-93 affects the growth and angiogenesis of human glioblastoma cells [16]. Currently, an increasing number of miR-93 target genes have been identified, such as FUS-1, LATS2, ITGB8, PTEN, TP53INP1 and DAB2, suggesting miR-93 may differentially affect the behaviours of tumours [10, 14, 16–19]. However, there are few reports of the role of miR-93 in HNSCC, particularly its relationship with HNSCC prognosis.

Accordingly, to further clarify the role of miR-93 in HNSCC, we evaluated miR-93 expression in a large number of HNSCC tissue samples using miRNA in situ hybridisation. Subsequently, we determined whether miR-93 expression was correlated with clinicopathological parameters and prognosis in patients with HNSCC using statistical methods.

## Materials and methods

### Patients and tissue specimens

A total of 103 archival paraffin HNSCC tissues were selected from patients with HNSCC who underwent surgeries in the Department of Otolaryngology of Xiangya Hospital in Central South University from January 2002 to November 2008. All paraffin samples were provided by the Department of Pathology of Xiangya Hospital in Central South University and were confirmed by haematoxylin–eosin (HE) staining. No patients recruited into the present study received any treatment prior to surgery. The patients were between 27 and 80 years old and included 99 males and 4 females. Of the cases, 26 cases were supraglottic, 57 were glottic, 10 were hypopharyngeal carcinomas and 10 were oral cancer located in buccal mucosa. For pathological grading (WHO, 1998), 16 were staged as well differentiated (G_1_), 21 as moderately differentiated (G_2_) and 66 as poorly differentiated (G_3_). Thirty-nine patients with lymph node metastasis were validated by conventional postoperative pathological examinations, and one case with distant metastases was confirmed by imaging evaluation. TNM staging was determined based on the Union for International Cancer Control (UICC, 2002). The clinical tumour stage distribution was stage I in 15 patients (T_1_N_0_M_0_ 15 cases), stage II in 26 patients (T_2_N_0_M_0_ 26 cases), stage III in 39 patients (T_3_N_0_M_0_ 19 cases, T_1_N_1_M_0_ 1 case, T_2_N_1_M_0_ 5 cases and T_3_N_1_M_0_ 14 cases) and stage IV in 23 patients (T_2_N_2_M_0_ 5 cases, T_3_N_2_M_0_ 7 cases, T_3_N_3_M_0_ 1 case, T_3_N_1_M_1_ 1 case, T_4_N_0_M_0_ 4 cases, T_4_N_1_M_0_ 3 cases and T_4_N_2_M_0_ 2 cases). A retrospective chart review was performed to retrieve the clinicopathological data (Table 1). Among the 103 paraffin HNSCC tissues, 16 cases with non-cancer epithelium were found in the same section. Due to the small number of control adjacent non-cancer tissues in the above paraffin tissues, another 17 pairs of fresh HNSCC tissues and their corresponding adjacent tissues were also collected and stored at −80 °C for the quantitative real-time PCR (qRT-PCR) detection.

**Table 1:** Clinicopathological factors in 103 patients with HNSCC

Factors	Number	Percentage
Age		
<58	47	45.6
≥58	56	54.4
Gender		
Male	99	96.1
Female	4	3.9
Tumour grade		
G_1_	16	15.5
G_2_	21	20.4
G_3_	66	64.1
Tumour site		
Supraglottic	26	25.2
Glottic	57	55.3
Hypopharyngeal	10	9.7
Oral	10	9.7
T classification		
T_1_	16	15.5
T_2_	36	35.0
T_3_	42	40.8
T_4_	9	8.7
Lymph node involvement		
N_0_	64	62.1
N_1_	24	23.3
N_2_	14	13.6
N_3_	1	1.0
Clinical stage		
I	15	14.6
II	26	25.2
III	39	37.9
IV	23	22.3

### Follow-up

The main methods of follow-up included outpatient review, telephone and visits. Metastasis was diagnosed by clinical examination, imaging evaluation and pathological studies. Termination of follow-up was due to deaths caused by HNSCC, and if the death caused by HNSCC was not observed until the last follow-up time, the data were considered censored data. A total of 99 of 103 patients were followed up. Four patients were lost because of change of contact or address. The follow-up period ranged from the day of surgery to the last follow-up time, and the longest follow-up time was 60 months.

### MiRNA in situ hybridisation

In situ hybridisation (ISH) for miR-93 was performed in 103 samples of HNSCC tissues, in which 16 cases of non-cancer epithelium were also observed in the same paraffin section. Paraffin-embedded, formalin-fixed tissues were cut into 4-μm sections. Locked Nucleic Acid (LNA) hybridisation probes double labelled with digoxigenin (DIG) complementary to human mature miR-93 were purchased from Exiqon (Denmark). The sequence of the miR-93 probe is 5′-CTA CCT GCA CGA ACA GCA CTT TG-3′. Briefly, the sections were deparaffinised in two consecutive xylene baths for 15 min each, followed by 5 min each in serial dilutions of ethanol (99.9, 96 and 70 %) and 5 min in phosphate-buffered saline (PBS) solution. The slides were then digested with 15 μg/ml proteinase K at 37 °C for 10 min, washed twice with PBS, gradually dehydrated in increasing concentrations of ethanol (70, 96 and 99.9 %) for 1 min each and air-dried completely. The LNA probe (40 nM) was denatured at 90 °C for 4 min and then hybridised for 1 h at 55 °C. A stringency wash was then performed by rinsing the slides at 55 °C once in 5× saline sodium citrate (SSC) buffer, twice in 1× SSC and twice in 0.2× SSC; at room temperature in 0.2× SSC; and finally in PBS-T (0.1 % Tween-20 in PBS). The slides were placed in blocking solution (0.1 % Tween, 2 % sheep serum and 1 % bovine serum albumin (BSA) in PBS) for 15 min at room temperature. An anti-digoxigenin antibody (Roche, Switzerland) at a 1:800 dilution in diluent solution (0.05 % Tween, 1 % sheep serum and 1 % BSA in PBS) was applied to the slides for 60 min at room temperature, followed by three washes in PBS-T. Nitroblue tetrazolium and 5-bromo-4-chloro-3-indolyl phosphate (NBT/BCIP) solution reagent was used to detect the signal. The slides were rinsed twice in KTBT buffer (50 mM Tris–HCl, 150 mM NaCl and 10 mM KCl), dehydrated in increasing concentrations of ethanol and mounted using a mounting medium. The slides were then scored by two pathologists independently using a transmission light microscope. The staining intensity and proportion of positive cells were scored independently. According to previous evaluation criteria of ISH reported by Lv and He [20, 21], the following parameters were recorded: (1) the staining intensity was scored as 0 (−), 1 (+, light blue), 2 (++, moderate blue) and 3 (+++, dark blue) and (2) the proportion of positive cells was scored as 0 (0 %), 1 (1–10 %), 2 (11–50 %) and 3 (51–100 %). The final scores = (1) × (2), and low expression was defined as a score from 0 to 5, whereas high expression was defined as a score from 6 to 9.

### Cell culture

The HNSCC cell lines M2, M4, Tu212 and Tu686 were kindly provided by Dr. Zhuo (Georgia) Chen (Emory University Winship Cancer Institute, Atlanta, GA). All cell lines were maintained in Dulbecco’s modified Eagle’s medium (DMEM)/F12 medium (1:1) (HyClone, USA) containing 10 % foetal bovine serum (FBS) (Gibco, USA), 100 IU/ml penicillin and 100 IU/ml streptomycin (Gibco, USA) and cultured in a humidified 5 % CO_2_ incubator at 37 °C. Compared to the poorly metastatic HNSCC cell lines (Tu686 and Tu212), M2 and M4 showed the highest metastatic ability in vivo (in nude mice orthotopically transplanted tumour model M2 with 50 % cervical lymph node metastasis, M4 with 100 % cervical lymph node metastasis and 75 % lung metastasis, respectively) [22].

### MiRNA isolation and quantitative real-time PCR

qRT-PCR for miR-93 was performed in 4 HNSCC cell lines and 17 pairs of fresh HNSCC tissues (including HNSCC tissues and their corresponding adjacent tissues). The miRNA was extracted from the tissues and cells using an RNeasy Mini Kit (Qiagen, USA). The qRT-PCRs were performed on a ViiA™ 7 Real-Time PCR System (Applied Biosystems, USA) using the All-in-One™ miRNA qRT-PCR Detection Kit (GeneCopoeia, USA) according to the manufacturer’s instructions. U6 small nuclear RNA (snRNA) was used as an internal control. Data were normalised using the U6 snRNA and the fold change of miR-93 was calculated using the 2^−ΔΔCt^ method [16]. The data are the average of three qRT-PCR replicates for each sample from three biological repeats.

### Statistical analysis

Statistical significance between the expression of miR-93 and clinicopathological parameters was analysed with the *χ* ^2^ test. Differences in miR-93 expression between the HNSCC cell lines and normal laryngeal mucosa tissues were compared with Student’s *t* test. Overall survival curves were plotted according to the Kaplan–Meier method, with the log-rank test used for comparison. The Cox proportional hazards model was used in the subsequent univariate analysis and multivariate analysis to identify the factors that were independent indicators for prognosis. The statistical analyses were performed using the SPSS 19.0 software. The results were regarded as statistically significant at *P* ≤ 0.05.

## Results

### ISH results of miR-93

**Fig. 1:**
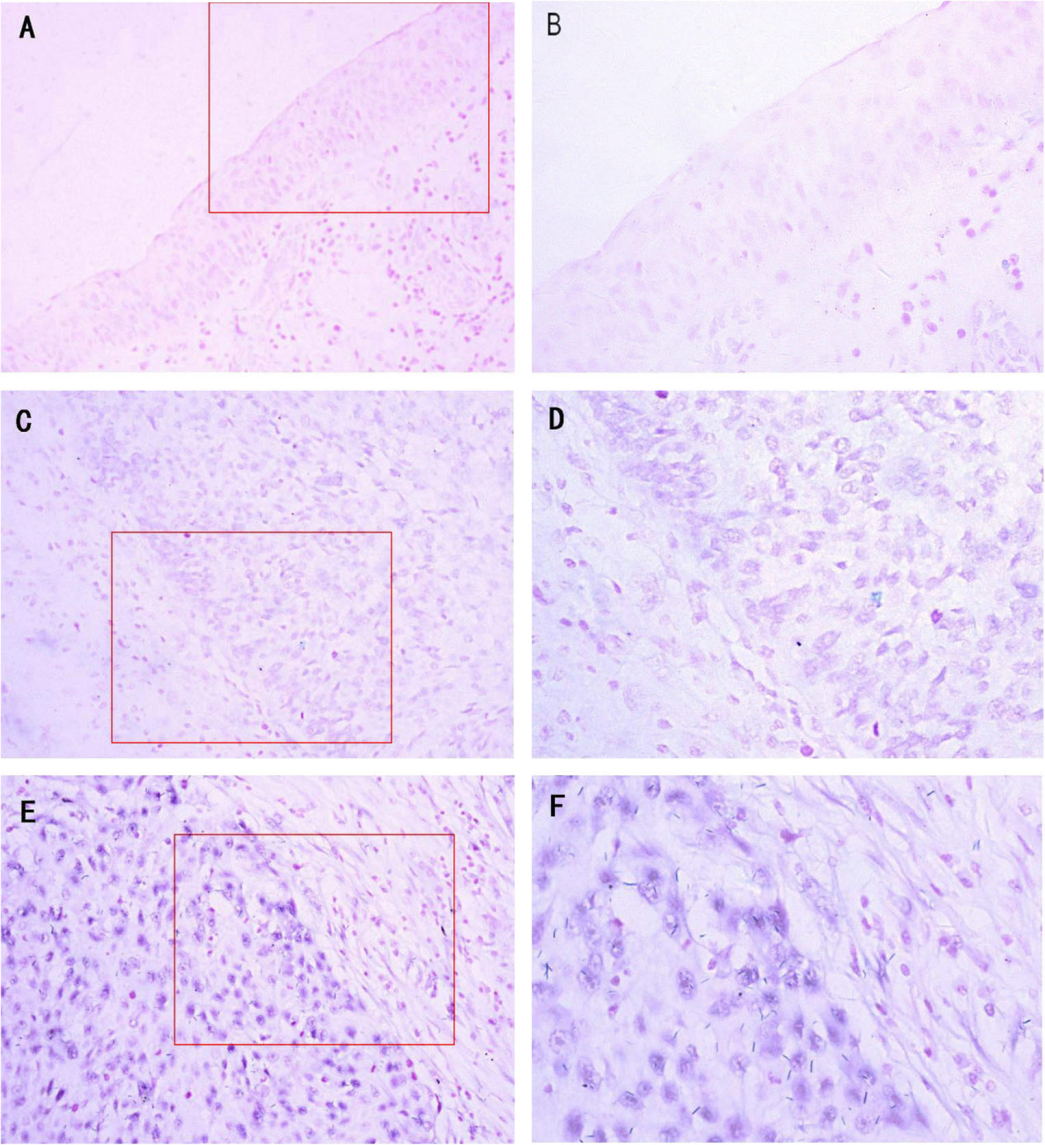
Representative in situ hybridisation for miR-93 expression in primary human HNSCC tissues and non-cancer epithelium. **a**, **b** Expression of miR-93 in non-cancer epithelium. **c**, **d** Low expression of miR-93 in HNSCC tumour tissues. **e**, **f** High expression of miR-93 in HNSCC tumour tissues (original magnification ×200 in **a**, **c** and **e**; ×400 in **b**, **d** and **f**, corresponding to the *red rectangle location* in **a**, **c** and **e**)

To examine the clinical relevance of miR-93 in HNSCC, its expression was analysed by ISH in 103 HNSCC tissues. Among these HNSCC tissues, 16 cases with non-cancer epithelium were observed in the same paraffin section under a microscope. Positive miR-93 was blue stained and predominantly observed in the cytoplasm of HNSCC cells (Fig. 1). Among all HNSCC samples analysed, 66 cases (64.08 %) demonstrated low miR-93 expression (Fig. 1c, d) and 37 cases (35.92 %) demonstrated high miR-93 expression (Fig. 1e, f). The positive rate was 92.23 % in HNSCC tissues, while miR-93 expression was not detected or just mildly expressed in all 16 cases of non-cancer epithelium tissues (Fig. 1a, b).

### Association between miR-93 expression and clinicopathological factors

The clinicopathological factors of the HNSCC patients were analysed in relation to the miR-93 levels using the *χ* ^2^ test (Table 2). Interestingly, the increased expression of miR-93 correlated well with tumour T classification (*χ* ^2^ = 12.711, *P* < 0.001), lymph node metastasis (*χ* ^2^ = 11.446, *P* = 0.001) and clinical stage (*χ* ^2^ = 10.513, *P* = 0.001). However, no significant relationship was observed for age (*χ* ^2^ = 0.603, *P* = 0.437), gender (*χ* ^2^ = 0.000, *P* = 1.000), tumour grade (*χ* ^2^ = 2.520, *P* = 0.112) or tumour site (*χ* ^2^ = 0.047, *P* = 0.829).

**Table 2:** Association between miR-93 expression and clinicopathological factors in patients with HNSCC

Factors	Number	MiR-93 expression		*χ* ^2^	*P* value
		High	Low		
Age					
<58	47	15	32	0.603	0.437
≥58	56	22	34		
Gender					
Male	99	36	63	0.000	1.000
Female	4	1	3		
Tumour grade					
G_1_ + G_2_	37	17	20	2.520	0.112
G_3_	66	20	46		
Tumour site					
Glottic	57	21	36	0.047	0.829
Others	46	16	30		
T classification					
T_1_ + T_2_	52	10	42	12.711	<0.001*
T_3_ + T_4_	51	27	24		
Lymph node involvement					
Positive	39	22	17	11.446	0.001*
Negative	64	15	49		
Clinical stage					
I + II	41	7	34	10.513	0.001*
III + IV	62	30	32		

*P* values were determined by the *χ* ^2^ test

*P ≤ 0.05 was considered to be statistically significant

### MiR-93 expression in fresh HNSCC tissues and HNSCC cell lines

**Fig. 2:**
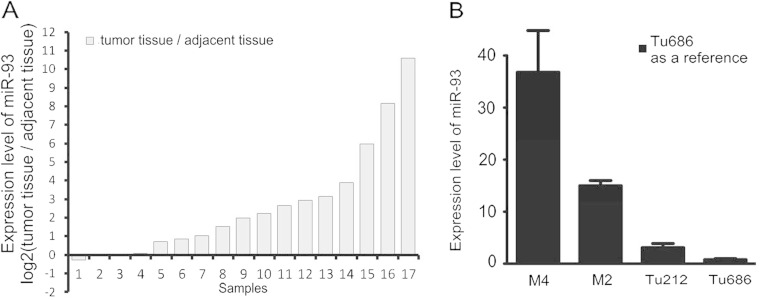
MiR-93 levels in HNSCC fresh tissues and cell lines. **a** MiR-93 levels in tumour tissues and their adjacent tissues. Higher miR-93 expression levels were observed in most HNSCC tissues. **b** MiR-93 levels in HNSCC cell lines. Cell lines M2 and M4 with the highest metastatic ability showed the higher miR-93 expression levels, while Tu686 and Tu212 with low metastatic capacity presented a relatively low miR-93 expression level. The data are the average of three qRT-PCR replicates for each sample from three biological repeats

Due to the small number of non-cancer tissues as a control in the above paraffin tissues, another 17 pairs of fresh HNSCC tissues and their adjacent tissues were also selected to assay miR-93 expression. Compared to that in adjacent tissues, miR-93 expression was significantly increased in HNSCC tissues (Fig. 2a), which was consistent with the result from the ISH assay in paraffin tissues. Meantime, based on the positive results between miR-93 expression and metastasis in the above ISH experiment, we validated the differential expression of miR-93 expression via qRT-PCR in the cell level, which included poorly metastatic cell lines (Tu686 and Tu212) and the most highly metastatic cell lines (M2 and M4). As shown in Fig. 2b, cell lines M2 and M4 with the highest metastatic ability showed the higher miR-93 expression levels, while Tu686 and Tu212 with low metastatic capacity presented a relatively low miR-93 expression level.

### Survival analysis

**Fig. 3:**
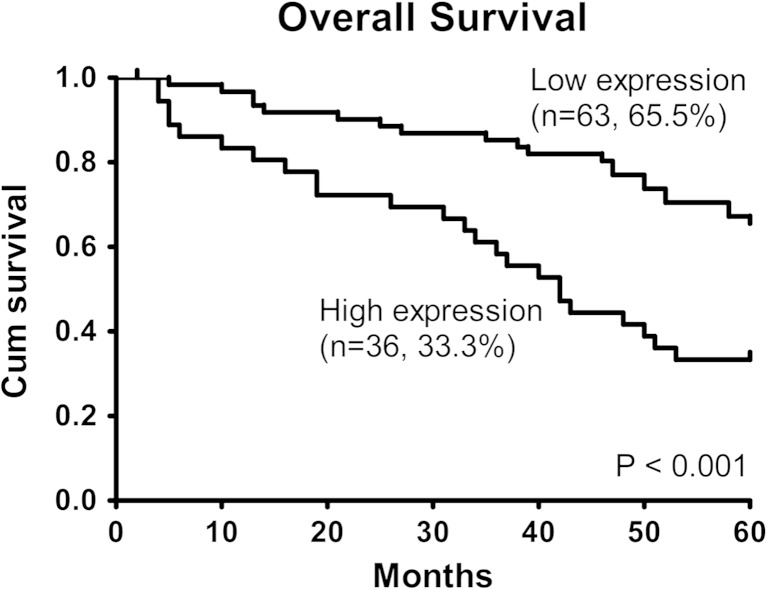
Survival in patients with HNSCC in relation to the miR-93 level. The overall survival was assessed using the Kaplan–Meier method, and the survival difference between groups was compared using the log-rank test (*χ* ^2^ = 12.648, *P* < 0.001). The overall survival of the high miR-93 expression group was significantly lower than that of the low miR-93 expression group (33.3 vs. 65.5 %)

Ninety-nine patients with intact follow-up information were included in the survival analyses. The overall survival rate of 5 years in the 99 patients was 53.6 %. Based on the miR-93 level, 99 patients with HNSCC were subdivided into 2 groups as follows: 63 patients in the low-expression group and 36 patients in the high-expression group. For the overall survival curves using Kaplan–Meier analysis, patients in the high miR-93 expression group (33.3 %, 38.5 ± 3.4 months) had significantly poorer prognosis than those in the low miR-93 expression group (65.5 %, 51.8 ± 2.0 months) (log-rank test: *χ* ^2^ = 12.648, *P* < 0.001, Fig. 3). Furthermore, to estimate the independent prognostic factors for HNSCC, univariate and multivariate analyses were performed using the Cox proportional hazards model. As summarised in Table 3, positive lymph node metastasis (relative risk (RR) = 0.359, 95 % confidence interval (CI) 0.143–0.901, *P* = 0.029) and increased miR-93 expression (RR = 0.515, 95 % CI 0.265–1.001, *P* = 0.050) were independent risk factors affecting the overall survival of patients with HNSCC.

**Table 3:** Univariate and multivariate analyses for overall survival (Cox proportional hazards regression model)

Factors	Univariate analysis		Multivariate analysis	
	95%CI	*P* value	95%CI	*P* value
Age (<58/≥58)	0.634–2.042	0.665		
Gender (male/female)	0.173–1.806	0.331		
Tumour grade (G_1_ + G_2_/G_3_)	0.497–1.685	0.775		
Tumour site (glottic/others)	1.629–5.565	<0.001*	0.719–2.988	0.292
T classification (T_1_ + T_2_/T_3_ + T_4_)	0.165–0.602	<0.001*	0.448–2.546	0.883
Lymph node metastasis (+/−)	0.071–0.274	<0.001*	0.143–0.901	0.029*
Clinical stage (I + II/III + IV)	0.051–0.332	<0.001*	0.077–1.371	0.126
MiR-93 expression (high/low)	0.200–0.649	0.001*	0.265–1.001	0.050*

All the clinicopathological factors listed in the table were included in the univariate and multivariate analyses

*95%CI* 95 % confidence interval

*P ≤ 0.05 was considered to be statistically significant

## Discussion

MiR-93 is a miRNA from the miR-106b-25 cluster, located in intron 13 of the host gene MCM7 at chromosome 7q22, and plays a regulatory role in a variety of malignant tumours [9]. However, the exact role of miR-93 remains unclear in HNSCC. In the present study, it was shown that miR-93 was differentially expressed in HNSCC tissues and cells and that the expression of miR-93 was related to T classification, lymph node metastasis, clinical stage and prognosis in HNSCC patients, which suggests that miR-93 may play an oncogenic role in HNSCC.

Verifying miR-93 expression in a large number of HNSCC tissues using miRNA in situ hybridisation has not yet been reported. In this study, we investigated the expression of miR-93 in 103 HNSCC tissues and found that miR-93 was positively expressed in 92.23 % of HNSCC tissues, whereas miR-93 was not detected or mildly expressed in non-cancer epithelium and adjacent HNSCC tissues. We detected miR-93 expression in four HNSCC cell lines using qRT-PCR and found that miR-93 was overexpressed in HNSCC cells. Moreover, miR-93 was found overexpressed in many other tumours, such as non-small cell lung cancer, oesophageal cancer, gastric cancer, hepatocellular carcinoma, breast cancer, cervical cancer, ovarian cancer, myeloid leukaemia, lymphoma, basal cell carcinoma, etc. [13, 23–31]. However, few studies showed that the expression of miR-93 was decreased in colon cancer and glioblastoma [32, 33]. This controversial result may be explained by the different histological types of tumours, leading to different expression levels of miR-93.

We determined the correlation between miR-93 expression and clinicopathological characteristics in patients with HNSCC and found that miR-93 expression was closely related to the T classification, lymph node metastasis and clinical stage of HNSCC. The positive correlation between miR-93 and lymph node metastasis is the major finding in our current investigation, and similar results are also reported in other human malignancies containing nasopharyngeal carcinoma and gastric cancer [25, 34]. These findings indicate the potential role of miR-93 in cancer metastasis. Previous studies have demonstrated that miR-93 regulates cancer metastasis via regulating different metastasis genes and pathways. In breast cancer, miR-93 was involved in the epithelial mesenchymal transition (EMT) by inhibiting the expression of Smad7 and activating the TGF-β signalling pathway [35]. Additionally, miR-93 promoted the tube formation of endothelial cells by suppressing LATS2 in vitro and promoted angiogenesis and lung metastasis in vivo [14, 35]. In glioblastomas, miR-93 promoted the growth, migration and tube formation of endothelial cells by inhibiting the expression of ITGB8, which is a primary receptor of extracellular matrix proteins and regulates the adhesion between cells and the ECM [16]. These findings suggest that miR-93 contributes to invasion and metastasis via regulating diverse metastasis-associated mechanisms, such as inducing EMT, promoting angiogenesis and disrupting adhesion between cells and the ECM.

The relationship between miR-93 and prognosis in patients with HNSCC has not been studied. Based on the Kaplan–Meier survival analysis and log-rank test, our data showed that the 5-year overall survival rate of patients with high miR-93 expression was significantly lower than that of patients with low miR-93 expression (33.3 vs. 65.5 %). The multivariate Cox proportional hazards regression analysis showed that lymph node metastasis and the miR-93 expression level were independent prognostic factors for patients with HNSCC. These results show that miR-93 expression was negatively correlated with the prognosis of HNSCC patients, which is consistent with other studies in lung squamous cell carcinoma, gastric cancer and ovarian cancer [28, 36, 37]. Overall, miR-93 expression and lymph node metastasis are highly significant for the prognosis of patients with malignant tumours, which may contribute to the most suitable clinical management strategy decision in HNSCC patients.

In conclusion, analysing miR-93 expression and its relationship to clinicopathological factors and prognosis, our study found that elevated miR-93 expression was related to T classification, lymph node metastasis, clinical stage and poor prognosis in patients with HNSCC. Moreover, it was an independent prognostic factor in patients with HNSCC, which indicates that miR-93 may be an important molecular marker for assessing lymph node metastasis and prognosis in patients with HNSCC. Our next study will focus on validating whether miR-93 influences biological behaviours, such as metastasis in HNSCC in vitro, and we will further explore the molecular regulatory mechanisms related to miR-93, which may lead to miR-93 becoming a new target for the molecular-targeted therapy of HNSCC in the future.
